# Smoking characteristics of Polish immigrants in Dublin

**DOI:** 10.1186/1471-2458-8-428

**Published:** 2008-12-31

**Authors:** Zubair Kabir, Vanessa Clarke, Sheila Keogan, Laura M Currie, Witold Zatonski, Luke Clancy

**Affiliations:** 1 Research Institute for a Tobacco Free Society (RIFTFS), The Digital Depot, Thomas Street, Dublin, Ireland; 2 Department of Cancer Epidemiology & Prevention, Cancer Center & Institute of Oncology, 5 Roentgena Str, 02-781 Warsaw, Poland

## Abstract

**Background:**

This study examined two main hypotheses: a) Polish immigrants' smoking estimates are greater than their Irish counterparts (b) Polish immigrants purchasing cigarettes from Poland smoke "heavier" (≥ 20 cigarettes a day) when compared to those purchasing cigarettes from Ireland. The study also set out to identify significant predictors of 'current' smoking (some days and everyday) among the Polish immigrants.

**Methods:**

Dublin residents of Polish origin (n = 1,545) completed a previously validated Polish questionnaire in response to an advertisement in a local Polish lifestyle magazine over 5 weekends (July–August, 2007). The Office of Tobacco Control telephone-based monthly survey data were analyzed for the Irish population in Dublin for the same period (n = 484).

**Results:**

Age-sex adjusted smoking estimates were: 47.6% (95% Confidence Interval [CI]: 47.3%; 48.0%) among the Poles and 27.8% (95% CI: 27.2%; 28.4%) among the general Irish population (p < 0.001). Of the57% of smokers (n = 345/606) who purchased cigarettes solely from Poland and the 33% (n = 198/606) who purchased only from Ireland, 42.6% (n = 147/345) and 41.4% (n = 82/198) were "heavy" smokers, respectively (p = 0.79). Employment (Odds Ratio [OR]: 2.89; 95% CI: 1.25–6.69), lower education (OR: 3.76; 95%CI: 2.46–5.74), and a longer stay in Ireland (>24 months) were significant predictors of current smoking among the Poles. An objective validation of the self-reported smoking history of a randomly selected sub-sample immigrant group, using expired carbon monoxide (CO) measurements, showed a highly significant correlation coefficient (r = 0.64) of expired CO levels with the reported number of cigarettes consumed (p < 0.0001).

**Conclusion:**

Polish immigrants' smoking estimates are higher than their Irish counterparts, and particularly if employed, with only primary-level education, and are overseas >2 years.

## Background

Globalization and industrialization have both led to migration across geographical borders, and the recent merge of 27 European countries is no exception. The Republic of Ireland has experienced a cultural revolution since 2002, and is home to hundreds of thousands of migrants from all across the globe and from Eastern Europe in particular constituting some 12.5% of the total population [1]. The Polish immigrants constitute a large segment of the immigrant population in the Republic. Official statistics of 2006 report just over 63,000 Polish immigrants in Ireland but the unofficial figure is much higher and may be 200,000 [1].

Over thirty-five percent [35.3%] overall adult smoking prevalence was reported in Poland in 2004 [2]. A recent report in Ireland reported an overall 29% adult (≥ 18 years of age) smoking prevalence in 2007 [3]. Evidence suggests that new environments influence a change in social behavior and apparently it takes at least 4/5 years before acculturation occurs among recent immigrants [4,5]. There is limited evidence to date characterizing the profile of smoking behavioral patterns in an immigrant population.

Poland is an EU member and its citizens can now enter Ireland freely after the 2002 enlargement. Therefore, the Polish immigrants studied are mainly recent immigrants. The change in economic circumstances and increased disposable income are likely to influence smoking patterns. Also the ease with which immigrants may travel between Poland where cigarettes are cheap and Ireland where cigarettes are dear probably influences cigarette consumption. In the context of such a background, we examined the following two hypotheses:

1) Polish immigrants' smoking estimates are greater than their Irish counterparts

2) Polish immigrants purchasing cigarettes from Poland smoke "heavier" (≥ 20 cigarettes a day) when compared to those purchasing cigarettes from Ireland.

## Methods

### Study setting

Dublin City Centre, Ireland

#### Study population

An advertisement in a local Polish lifestyle magazine SOFA was posted in the first quarter of 2007. Also 10 Polish interviewers were recruited, trained and posted, wearing red T-shirts, at a busy intersection of the Dublin city area [where numerous Polish shops are located] over a period of 5 weekends from July 7^th ^to August 4^th ^in 2007. A supervisory research team from RIFTFS also visited the study site on a regular basis. Finally, 1,545 Polish immigrants completed the questionnaire in Polish over the 5-week study period.

#### Inclusion criteria

The Polish interviewers recruited the study participants who answered "yes" to the following two questions:

a) Are you Polish by birth and live in Ireland?

b) Are you interested in taking part in a lifestyle survey?

#### Data collection

The immigrants completed an interviewer-administered, 20-item questionnaire in Polish. The original Polish questionnaire was translated into English, modified, back-translated into Polish and approved by a Polish research team before use. All smoking-related information was collected, in addition to demographic and socio-economic characteristics. Additional files 1 and 2 show the Polish and the English questionnaires, respectively. For an objective validation of the quantity of cigarettes consumed, expired CO levels (in parts per million [ppm]) were also measured in a random sub-sample of the Polish survey population using the Micor Medical Micro CO meter (Micor, Kent, UK) [6]. We also asked questions on quit attempts and intentions.

The following smoking-related information was mainly collected (see additional file 1 and additional file 2):

• How often do you currently smoke? [everyday; someday; not at all]

• Have you ever smoked daily for six months at least? If yes, how old were you when you started to smoke daily?

• If you do not currently smoke, how long has it been since you last smoked?

• If you do not currently smoke, what age were you when you quit?

• If currently smoke, how many cigarettes do you usually smoke per day?

• From where do you usually get your cigarettes? [Ireland; Poland; both]

• How much do you usually pay for a pack of 20 cigarettes?

The Irish Office of Tobacco Control (OTC) monitors cigarette smoking prevalence and behaviour nationwide. The data set is compiled from a monthly, quota survey conducted by telephone omnibus. The data consists of a collection of 1,000 responses per month from the Irish population over 15 years of age [7]. However, for the present study OTC data on individuals residing in Dublin alone during July and August 2007 were analyzed. This numbered 484 individuals. Such a procedure was adopted to comply with the internal consistency of the Polish survey, which was conducted in Dublin between July and August 2007. We present unweighted OTC data, which are almost similar to the weighted sample. Although OTC questions were not identical to the Polish survey and the methodology was also different for these two surveys, the current smoking prevalence estimates were based on almost similar set of questions ("How often do you currently smoke: everyday; someday; not at all?" in the Polish survey, and "Do you smoke one or more cigarettes per week?" for the OTC survey). Details of the OTC survey can be accessed at the OTC website .

### Statistical analyses

#### a) For smoking prevalence

Both "some days" and "everyday" smokers were included to estimate "current" smoking prevalence among the Polish immigrants as is done also in the OTC survey. Only those with complete information on smoking status were included for the smoking prevalence estimates (n = 1,375). The estimates are age-standardized to the general Polish age structure in Ireland for the census year 2006, using Rothman's EpiSheet [8]. Also, the OTC data are age-standardized to the general Irish age-structure for the census year 2006. However, modeled smoking prevalence rates were computed using Proc Logistic procedure of SAS (version 9.1) for both the survey population, to allow comparison and simultaneously adjust for age and sex [9]. Because of the modeled adjusted smoking prevalence estimates were almost similar to the age-standardized smoking estimates, only the modeled adjusted smoking estimates are presented (in table 1). The mean differences in smoking prevalence estimates between the two populations were also compared, using Proc ttest procedure in SAS software [9]. 95% confidence intervals (CI) for all smoking categories were computed.

**Table 1:** Adjusted smoking prevalence estimates (%) in the Polish immigrants (Pole survey) and in the general Irish population in Dublin (OTC survey), July–August 2007

	**POLE Survey (n = 1,375) %** **(95% CI)**	**OTC Survey (n = 484) %** **(95% CI)**	**'t' test (mean difference)**
**Overall (age-sex adjusted)**	47.6 (47.3; 48.0)	27.8 (27.2; 28.4)	p < 0.001
**Gender (age-adjusted)**			
Males	50.9 (50.5; 51.3)	27.4 (26.5; 28.2)	p < 0.001
Females	39.8 (39.5; 40.2)	28.3 (27.6; 29.0)	p < 0.001
**<19 year olds**			
Males	20.0 (6.0; 66.8)	5.9 (0.6; 61.3)	p < 0.001
Females	8.3 (0.9; 77.7)	12.5 (2.4; 64.7)	p < 0.001
**19–40 year olds**			
Males	51.6 (35.6; 74.8)	42.5 (18.1; 99.9)	p < 0.001
Females	40.6 (20.7; 79.7)	32.4 (15.6; 67.2)	p < 0.001
**>40 year olds**			
Males	50.0 (41.8; 59.8)	16.4 (11.0; 24.5)	p < 0.001
Females	41.1 (29.7; 57.2)	26.9 (19.7; 36.7)	p < 0.001

#### b) Bivariable and Multivariable Logistic Regression analyses

Bivariable analyses (categorical) were performed to predict the co-variates contributing to current smoking among the Polish immigrants versus those "not" currently smoking (this includes both never and former smokers). Tests of significance were assessed using Pearson χ^2 ^tests. To identify significant predictors of current smoking, backward elimination method of multivariable logistic regression modeling was performed, using SAS statistical software.

Finally, multivariable logistic regression analyses were performed to test the null hypothesis that there were no significant differences between purchase of cigarettes from Poland and heavy smoking. A "heavy" smoker was defined as an individual smoking ≥ 20 cigarette a day. Adjusted "heavy" smoking prevalence rates for the two categories [those purchasing cigarettes solely from Poland and those solely from Ireland] examined were estimated and tested for any significant differences [9].

#### c) Objective validation of the quantity of cigarettes consumed

Expired CO levels (ppm) were measured in a random sub-sample of 142 of the surveyed Polish population. However, information on the quantity of cigarettes smoked was available only for 90 individuals of those sampled for breath CO level measurements. Pearson correlation coefficient tests and inter-quartile range (IQR) of CO/ppm of the 90 individuals were assessed for objective validation, similar to a recent report [6].

## Results

A total of 1,545 Polish immigrants were surveyed in Dublin. Among the population surveyed (n = 1,545), 905 (58.6%) were males, 547 (35.4%) were females, and the remaining 93 (6%) had no information on gender. More than 70% of the survey population was between 20 and 40 years of age.

Table 1 shows that the modeled age-sex adjusted smoking prevalence estimates among these two surveyed population are almost similar to the age- standardized rates (47.6% and 27.8%, respectively). However, Poles have two-fold increased rates compared to the general Irish population smoking rates over the same period (p < 0.001); male Poles have significantly higher predicted smoking estimates (50.9%) than females (39.8%) which are significantly higher than their Irish counterparts (p < 0.001). 19–40 year olds have the highest smoking estimates across both the surveyed population (table 1). Almost 20% of the male Poles aged <19 years smoked, although the confidence intervals were wide (table 1). Irish females aged <19 years smoked significantly greater than their Polish counterparts (12.5% vs. 8.3%), respectively (p < 0.001). More than 20% of the Polish smokers surveyed are also heavy smokers compared to less than 10% among the general Irish population surveyed (p < 0.001).

Almost 50% of the current Polish smokers are also contemplating quitting and/or are actively planning to do. Also of interest, a greater proportion of the quitters are in 20–25 years of age, and the majority of the recent quitters (<5 years of abstinence) are females. Only 8% of the smokers in this study population ever sought medical advice on quitting smoking.

Table 2 shows the baseline characteristics of the Polish immigrants (n = 1,545). Table 3 shows the bivariable analyses between current smokers and those 'not' currently smoking (this includes both never-smokers and former smokers) across several demographic and socio-economic characteristics among the Polish immigrants. In general, all the variables studied are statistically significant (p < 0.05). However, on backward elimination method of multivariable logistic regression analyses, only three variables were identified as significant predictors of current smoking status among the Poles: education, occupation and the duration of stay in Ireland (table 4). Those employed were almost thrice as likely to smoke as the unemployed (adjusted OR: 2.89; 95% CI: 1.25–6.69); those with the lowest level of education (adjusted OR: 3.76; 95%CI: 2.46–5.74) are predisposed to smoking; and the shorter the stay in Ireland (<12 months) the less likely to smoke [adjusted OR: 0.61; 95%CI: 0.45–0.83] (table 4).

**Table 2:** Basic characteristics of the Polish immigrants (n = 1,545)

**Variables**	**No**	**%**
**Gender (n = 1,451)**		
Male	904	62.3
Female	547	37.7
**Age (n = 1,478)**		
<19 years	68	4.6
19–40 years	979	66.2
>40 years	431	29.2
**Marital status (n = 1,439)**		
Not single	981	68.2
Single	458	31.8
**Education (n = 1,532)**		
Primary	239	15.6
Secondary	878	57.3
Graduates/Higher	415	27.1
**Employment (n = 1,423)**		
Employed	1,237	86.9
Unemployed	186	13.1
**Salary (n = 1,313)**		
<€20,000	339	25.8
€20–39,999	875	66.7
>=€40,000	99	7.5
**Duration of stay (n = 1,517)**		
<1 year	540	35.6
1–2 years	238	15.7
>2 years	739	48.7
**Year of arrivals (n = 1,545)**		
2006 and earlier	892	57.7
2007	653	42.3
**Smoking status (n = 1,375)**		
Never Smokers	576	41.9
Former Smokers	154	11.2
Current Smokers	645	46.9

**Table 3:** Demographic and socio-economic characteristics between currently smoking and currently not smoking Polish immigrants (n = 1,375)

**Variables**	**Currently smoking (n = 645)**		**Currently not smoking (n = 730)**		**Pearson χ^2^**
	**No**	**%**	**No**	**%**	
**Gender (n = 1,295)**					**p = 0.0002**
Male	421	51.4	398	48.6	
Female	193	40.5	283	59.5	
**Age (n = 1,319)**					**p = 0.007**
<19 years	19	33.3	38	66.7	
19–40 years	395	45.6	472	54.4	
>40 years	208	52.7	187	47.3	
**Marital status (n = 1,287)**					**p = 0.03**
Not single	213	50.8	206	49.2	
Single	385	44.4	483	55.6	
**Education (n = 1,369)**					**p < 0.0001**
Primary	131	59.0	91	41.0	
Secondary	379	48.8	397	51.2	
Graduates/Higher	132	35.6	239	64.4	
**Employment (n = 1,271)**					**p = 0.047**
Employed	541	48.6	573	51.4	
Unemployed	63	40.1	94	59.9	
**Salary (n = 1,178)**					**p = 0.03**
<€20,000	119	41.2	170	58.2	
€20–39,999	401	50.1	400	49.9	
>=€40,000	44	50.0	44	50.0	
**Duration of stay (n = 1,356)**					**p < 0.0001**
<1 year	183	38.9	287	61.1	
1–2 years	106	48.2	114	51.8	
>2 years	345	51.8	321	48.2	
**Year of arrivals (n = 1,375)**					**p = 0.0007**
2006 and earlier	407	50.8	395	49.2	
2007	238	41.5	335	58.5	

**Table 4:** Significant predictors of current smokers among the Polish community in Dublin (backward elimination logistic regression modeling)

**Variables**	**Adjusted Odds Ratios (AOR)**	**95% Confidence Intervals (CI)**	
**Education**			p < 0.0001
Graduates/Higher	Reference		
Secondary	2.13	1.56–2.91	
Primary	3.76	2.46–5.74	
**Employment**			p = 0.01
Unemployed	Reference		
Employed	2.89	1.25–6.69	
**Duration of stay**			p = 0.002
>2 years	Reference		
1–2 years	0.97	0.68–1.38	
<1 year	0.61	0.45–0.83	

Of the reported smokers with full information (n = 606), 57% (n = 345) purchased cigarettes only from Poland, 33% (n = 198) purchased only from Ireland, and the remaining 10% (n = 63) purchased from both these countries or from a third country. Of the 345 Polish smokers, who purchased cigarettes solely from Poland, 147 [42.6%] were 'heavy' smokers compared to 41.4% (n = 82/198) 'heavy' smokers who purchased cigarettes from Ireland only, and this difference was not statistically significant (p = 0.79) [table 5]. On further adjustment for all the potential confounders, there was no significant difference in the "heavy" smoking prevalence rates between these two categories (table 5). Because of no significant differences observed in these two categories the adjusted odds ratios were not shown in table 5.

**Table 5:** Adjusted heavy smoking prevalence estimates among the Polish immigrant smokers by country of purchase of cigarettes (n = 607)

**Adjusted Smoking Prevalence**	**Purchase from Ireland**	**Purchase from Poland**	**'p' values**
Unadjusted	41.4%	42.6%	p = 0.79
Age-sex adjusted	42.5%	42.7%	p = 0.72
Adjusted for age, sex, Income	42.9%	47.8%	p = 0.17
Adjusted for age, sex, Occupation and income	43.7%	47.3%	p = 0.25
Adjusted for age, sex, Occupation, Income and Duration of stay in Ireland	43.9%	46.7%	p = 0.42
Adjusted for age, sex, Occupation, duration of stay, Income and marital status	42.1%	46.1%	p = 0.35
Adjusted for age, sex, Occupation, duration of stay Education, income, Marital status and Smoking age initiation	43.4%	45.5%	p = 0.52

Table 6 shows that the inter-quartile range is statistically significant (p = 0.001) between expired CO (in ppm) levels and the reported number of cigarettes consumed. A highly significant (p < 0.0001) correlation coefficient of 0.64 between CO (in ppm) and the reported quantity of cigarettes consumed was also observed (table 6).

**Table 6:** Two different validation methods of smoking history by measuring carbon monoxide (CO)/ppm in the breath of randomly selected Polish immigrants (n = 90)

**1) Inter-quartile Range of CO/ppm**	**Heavy smokers**		**Light smokers**		**p = 0.001**
	**No**	**%**	**No**	**%**	
2–9	3	8.8	20	35.7	
10–22	12	35.3	24	42.9	
23–63	19	55.9	12	21.4	
					
**2) Correlation between CO levels in ppm and the number of cigarettes smoked**					
r = 0.64	p < 0.0001				

## Discussion

This is a unique study characterizing the smoking profile of a large immigrant population who moved country as a consequence of the freedom of movement resulting from an enlargement of the EU at a time of unprecedented economic growth in the recipient country, Ireland. Most of the population shift took place over a short period of 2–4 years. It also took place at a time when air travel was very cheap with the result that it was feasible to commute a 1000 km for the cost of a packet of cigarettes in the host country. Further the number of Polish immigrants was such that it represented 5% of the total Irish population. The finding that Polish immigrants if employed but not educated to a graduate or post-graduate level are more likely to smoke in this situation seems likely to be linked to the easy availability of cheap cigarettes in their native country which they or their friends visited frequently.

Furthermore, such findings may provide additional insights into the economics of tobacco use among an immigrant population. Cigarette prices are very dear in Ireland when compared with cigarette prices in Poland €7.00 vs. €1.45 for a 20 pack, respectively. It is suggested that this "bargain" effect may be a potent inducement to smoke in the less well-educated relatively well-paid new immigrants. This study also showed that current smoking rates are almost two-fold higher among the Polish immigrants when compared to their Irish counterparts. One-fifth of the Polish smokers are also heavy smokers. Nevertheless, this study failed to show a statistical significant association of purchasing cigarettes from Poland and being a heavy smoker when compared to purchasing cigarettes solely from Ireland.

This study also showed that those Poles who arrived less than a year in Ireland were less likely to smoke. Such an observation might suggest the complex interaction between acculturation and health behavioral patterns of recent and past immigrants [4,5]. A very high smoking rate among the most productive age-groups (19–40 years) across both the population groups surveyed is a matter of concern for long-term productivity loss related to sustained tobacco use. The age-sex adjusted 47.6% overall smoking prevalence among the Polish immigrants is also substantially higher than the reported 35.3% overall adult smoking prevalence in Poland in 2004 [3,10]. We confirmed as in other studies that educational level attained was inversely related to smoking Employment status and salary being directly related to smoking is the opposite to what is seen in the Irish in Ireland and in the Poles in Poland where the less well-educated and economically handicapped are more likely to smoke [2,3].

### Study limitations and strengths

Because of the "selected" nature of the recruitment procedure, generalization of the study findings is less likely. However, the objective validation of smoking history, using expired CO measurements strengthens the self-reported smoking history. In addition, former (~10% of the survey population) were excluded for the estimation of adjusted current smoking prevalence rates, thereby, not further diluting the estimates.

### Public-health policy implications

The fact that the majority of the Polish immigrants in this study are educated and employed and have high income and yet have very high smoking rates signals that the determinants of smoking in immigrants may be different from the general population. The sudden and marked change in economic circumstances and increased disposable income seem to be stronger influences than education alone. Also, the ease and low cost with which immigrants may travel between Poland where cigarettes are cheap and Ireland where cigarettes are dear probably influences cigarette consumption. The need for smoking cessation services which take these findings into consideration is clear and probably also needs to address the cultural and gender aspects of smoking in an immigrant population. The fact that only 8% seek medical advice despite 50% wanting to quit suggest that the current cessation services available need to be adapted to address the special circumstances uncovered in this study.

## Conclusion

In conclusion, the study findings are unique despite methodological limitations, conducted in a real-life situation, and provide useful insights into the smoking behavior of an immigrant population, and this is particularly important in the context of the Irish Government's commitment towards a Tobacco Free Society [11]. Smoking rates and the levels of consumption of cigarettes are very high in Polish immigrants in Dublin and are much higher than in the comparable Irish population and are also much higher than the comparable population living in Poland. The majority of these immigrants smoke cigarettes bought in Poland where prices are much lower than in Ireland (€1.45. v €7.00). Being employed and lower educational status are strong predictors of current smoking. High earnings are not associated with lower smoking rates. Immigrants of longer domicile (>24 months) are more likely to be smokers. The impact of these population changes on tobacco control should be monitored. The study findings also indicated the need for an exploratory study surrounding the economics of tobacco use among an immigrant population, which seems to be different from the general population.

## Competing interests

The authors declare that they have no competing interests.

## Authors' contributions

LC conceived the study design, secured funding, liaised with the polish community and supervised the study and had a significant role in drafting; VC, SK and LMC contributed to data collection and piloting of the questionnaire; WZ contributed to the original design of the questionnaire, ZK did the analyses and prepared the original draft manuscript. The funding agency had no role in the study design, interpretation of results or decision to publish. All the authors contributed to the manuscript writing and have approved the final version. LC is the guarantor of this paper.

## Pre-publication history

The pre-publication history for this paper can be accessed here:



## Supplementary Material

Additional file 1 **Questionnaire 1**. Survey questionnaire in Polish. Click here for file

Additional file 2 **Questionnaire 2**. Survey questionnaire in English. Click here for file
