# Supplementary material for: Smoking characteristics of Polish immigrants in Dublin
Source: BMC Public Health. 2008 Dec 31;8:428. doi: 10.1186/1471-2458-8-428 (PMC2630949; doi:10.1186/1471-2458-8-428)
Supplement: Additional file 1 — Questionnaire 1. Survey questionnaire in Polish. [file 1471-2458-8-428-S1.doc]

**Badanie marketingowe rynku - kwestionariusz: Polonia w Irlandii ankieta PL001/07**

***Market research – questionnaire: Polish Community in IrelandPL001/07***

**Interviewer’s No. Respondent’s No. Starting time of interview**

**SECTION 1**

**1.1 Płeć *gender* Mężczyzna** *male*  **Kobieta** *female*

**1.2 Proszę podać swój rok urodzenia *Year of Birth***

**1.3 Jaki jest Pana(i) stan cywilny? *What is your current marital status?***

a) kawaler/panna *single*

b) konkubinat *cohabiting*

c) żonaty/zamężna *married*

d) w separacji *separated*

e) rozwiedziona *divorced*

f) wdowiec/wdowa *widowed*

**1.4 Jakie jest Pana (i) wykształcenie, tzn. jaką szkołę ukończył(a) Pan(i) jako ostatnią?**

*What is the highest level of education you have completed to date?*

a) niepełne podstawowe *some primary (not complete)*

b) podstawowe *primary or equivalent*

c) gimnazjum/zasadnicze zawodowe *intermediate/junior certificate or equivalent*

d) średnie (liceum, technikum) *secondary/leaving certificate or equivalent*

e) pomaturalne/policealne *diploma/certificate*

f) licencjat/magister *primary degree*

g) wyższe podyplomowe *postrgraduate/higher degrees*

**1.5 Zatrudnienie** *Which of the following descriptions best describes your current situation with regard to work?*

a) pracownik *Employee (including apprentice or Community Employment)*

b) prowadzę własną działalność *gospodarczą Self employed (not including farmer)*

c) rolnik *Farmer*

d) student studiów dziennych *Student Full time*

e) trening państwowy (FAS, Failte Ireland, itp.) *On state training scheme (FAS, Failte Ireland etc)*

f) bezrobotny, aktywnie szukający zatrudnienia *Unemployed, actively looking for a job*

g) przewlekła choroba lub niepełnosprawność *Long term sickness or disability*

h) prace domowe/opieka nad domem lub członkiem rodziny *Home duties /looking after the home or family*

i) emeryt(ka), proszę podać jaki był ostatni zawód przed emeryturą *Retired (please specify last occupation before retirement)*

j) inne *Other (please specify* …………

**1.6 Średnie dochody wszystkich członków rodziny ze wszystkich źródeł po odliczeniu podatku i PRSI.**

*What is your approximate level of net household income? This means the total income, after tax and PRSI, of ALL MEMBERS of the household. It includes ALL TYPES of income: income from employment, social welfare payments, child benefit, rents, interest, pensions etc. We would just like to know into which broad group the total income of your household falls.*

| Na tydzień *Per week* (€) | Na miesiąc *Per month* (€) | Na rok *Per year* (€) | Zaznacz *Tick* |
| --- | --- | --- | --- |
| Poniżej *Under* 193 | Poniżej *Under* 834 | Poniżej *Under* 10000 |  |
| 193 - 384 | 834 - 1667 | 10000-19999 |  |
| 385 – 575 | 1668 - 2500 | 20000-29999 |  |
| 576 - 767 | 2501 - 3333 | 30000-39999 |  |
| 768 - 959 | 3334 - 4167 | 40000-49999 |  |
| €960 lub więcej *or more* | 4168 lub więcej *or more* | 50000 lub więcej *or more* |  |

**1.7 W jakim kraju się urodziłeś(aś)?** *In what country were you born?*

a) Irlandia *Ireland*

b) Polska *Poland*

c) inny *other*

**1.8 Jeśli urodziłeś(aś) się w Polsce lub innym kraju, podaj miesiąc i rok przyjazdu do Irlandii** . *If born in Poland, when did you arrive in Ireland? (Month/Year)*

Miesiąc *month* Rok *year*

**1.9 Czy powracałeś(aś) od tego czasu do Polski z myślą stałego zamieszkania tam?** *Have you ever had returnem to Poland planning to stay back for good?*

a) tak *yes*

jeśli tak, ile razy i jak długo przebywałeś(aś) w Polsce *if so, please state how many Times and how long did you stay* …………….

b) nie *no*

**1.10 Jak długo planujesz mieszkać w Irlandii?** *In total, how long do you plan to live in Ireland?*

a) 0 – 6 miesięcy *0-6 mths*

b) 7 – 12 miesięcy *7-12 mths*

c) 1 – 1,5 roku 1-1,5 yrs

d) 1,5 – 2 lat *1,5-2 yrs*

e) więcej niż dwa lata *more than 2 yrs*

f) na stałe *for good*

**1.11 Jaki był powód Twojego przyjazdu do Irlandii?** *What was the reason of your arrival in Ireland?*

a) Ekonomiczno-zarobkowy *economical*

b) Edukacja/nauka *education/study*

c) Przyjazd do rodziny (do męża/żony, itp.) *joing family mamber*

d) Inne (podaj) *other (specify)*…….

**1.12 Czy jesteś zadowolony ze swojej pracy w Irlandii?** *Are satisfied with your employment conditions and your rights?*

a) Tak *Yes*

b) Nie *No*

**1.13 Czy w najbliższym czasie (3 – 6 mcy) rozważasz zmianę pracy/podjęcie nauki?**

a) Tak *Yes*  **Praca Nauka**

b) Nie *No*

**1.14 Czy był(a)byś zainteresowany rozwojem swojej kariery zawodowej w:** *Would you be interested in your professional career development in:*

a) Irlandii *Ireland*

b) Wielkiej Brytanii *UK*

c) Polsce *Poland*

d) USA

e) Kanada *Kanada*

f) Australia

g) Nowa Zelandia *New Zealand*

h) innym kraju UE lub na świecie *in another EU country or in the world* jakim *specify*

**1.15 Miejsce zamieszkania (Dublin – dzielnica lub County) np. Dublin 7 lub Co. Dublin, Co. Meath itp.** *My current place of residency (town,city,postal code if Dublin, Co.)*

**1.16 Czy posiadasz dostęp do Internetu? *Have you got Access to the Internet?***

a) TAK *Yes*  w domu/*at home*  pracy/*At work* w kafejce/*in Internet Café* Laptop PC

b) NIE *No*

**1.17 W jakim celu korzystasz z Internetu?** *What do you usually do on the Internet?*

a) Email

b) Szukam pracy *seeking Job*

c) Szukam mieszkania *Seeking accommodation*

d) Szukam … *Seeking ….*

e) Wiadomości *news*

f) Czat/Chat (nazwa *name*)

g) Randki *dating*

h) bilety lotnicze *airline tickets*

i) Inne *other*

**1.18 Czy oglądasz przekazy TV, wiadomości lub inne programy online / w Internecie?**

*Do you watch any online TV broadcasts?*

a) Tak *Yes TV Radio Inne Jakie: …………*

b) Nie *No*

Jak często? *How often?*

**1.19 Czy znasz jakieś polonijne serwisy w Internecie?** *Do you know any Polish Web sites abroad?*

a) gazeta.ie

b) polskagazeta.ie

c) polskidublin.com

d) dublinek.net

e) wyspa.ie

f) metoo.ie

g) inne (jakie, wymień) *other* …

**1.20 Z jakich w Polsce korzystasz?** *What Polish Web sites in Poland do you use?*

a) onet.pl

b) wp.pl

c) interia.pl

d) gazeta.pl

e) inne (jakie? wymień)… *other*

**1.21 Czy kupujesz lokalną prasę?** *Do you buy local newspapers?*

a) TAK, tylko polska prasę *Yes, only Polish* polska i irlandzka *Polish&Irish* Irlandzka *Irish*  inna *other*

b) NIE *No*

**1.22 Jakie tytuły kupujesz/czytasz?**

a) Polska Gazeta

b) Polski Express

c) Życie

d) Anons

e) Wyspa

f) SOFA

g) Nasz Głos

h) inne *other*

**1.23 Jak często kupujesz/czytasz?**

a) codziennie *everyday*

b) co tydzień *every week*

c) w weekendy *on weekends*

d) co dwa tygodnie *every two weeks*

e) co miesiąc *every month*

**1.24 Jakich informacji szukasz w prasie lub Internecie?** *What news do you look for online and in newspapers?*

a) ogłoszenia *classifieds*

b) Praca *jobs*

c) Zakwaterowanie *accommodation*

d) Szkoły językowe/edukacja *Language schools/education*

e) Wiadomości z Irlandii *news from Ireland*

f) Wiadomości z Polski  *News from Poland*

g) Wiadomości ze świata *World news*

h) publicystyka *journalism*

i) Inne/*other*

**1.25 Czego Ci brakuje w polonijnej prasie?** *What are you missing in Polish publications?*

Wymień *list* a) b) c)

**Spożycie tytoniu *Tobacco Use***

**2.1 Czy wypaliłeś w życiu 100 papierosów (5 paczek po 20szt.)?**

*Have you smoked at least 100 cigarettes (5 packs of 20) in your lifetime?*

1. Tak *Yes*
2. b) Nie *No* (jeśli nie 🡪 pomiń sekcje 2 o spożyciu tytoniu, *if No, then skip the rest of tobacco use section)*

**2.2 Czy kiedykolwiek paliłeś(aś) codziennie przez min. 6 miesięcy?** *Have you ever smoked daily for six months at least?*

1. Tak 🡪 jeśli tak, ile miałeś(aś) lat kiedy zacząłeś(aś) palić codziennie? _____ lat *Yes → If yes, how old were you when you started to smoke daily? ______ years*
2. Nie *No*

**2.3 Jak często obecnie palisz?** *How often do you currently smoke?*

1. Codziennie *Everyday*
2. Niekiedy *Somedays*
3. W ogóle nie palę *Not at all*

Jeśli odpowiedziałeś(aś) a) codzienie lub b) niekiedy, przejdź do pytań 6 – 12.

*If you answered a) Everyday or b) Somedays, please skip to question 6 through 12.*

Jeśli odpowiedziałeś(aś) c) W ogóle nie palę, przejdź do pytań 4 i 5. *If you answered c) Not at all, please answer question 4 and 5.*

**To jest sekcja tylko dla byłych palaczy *This section is for ex-smokers only***

**2.4** **Jeśli obecnie nie palisz**, ile minęło czasu odkąd ostatnio paliłeś(aś)?

***If you do not currently smoke****, how long has it been since you last smoked?*

1. Mniej niż miesiąc *Within the past month (anytime less than 1 month ago)*
2. 1 - 3 miesiące *Within the past 3 months (1 month but less than 3 months ago)*
3. 3 – 6 miesięcy *Within the past 6 months (3 months but less than 6 months ago)*
4. 6 – 12 miesięcy *Within the past year (6 months but less than 1 year ago)*
5. 1 – 5 lat *Within the past 5 years (1 year but less than 5 years ago)*
6. 5 – 10 lat *Within the past 10 years (5 years but less than 10 years ago)*
7. 10 i więcej lat *10 or more years ago*

**2.5 Jeśli obecnie NIE palisz, w jakim wieku byłeś(aś) kiedy rzuciłeś(aś) palenie?** …….*i****f you do not currently smoke****, what age were you when you quit? _____years old*

**TA SEKCJA JEST TYLKO DLA CZYNNYCH PALACZY The next section is for current smokers only**

**2.6 Jeśli obecnie palisz ile wypalasz dziennie?** ….. *If* ***you currently smoke,*** *how many cigarettes do you usually smoke per day? ____*

**2.7 Czy w przeciągu ostatniego roku lekarz lub doradca zdrowotny rozmawiał z Tobą o sposobach rzucenia palenia?** *In the past 12 months did a doctor or health professional discuss ways of giving up smoking with you?*

1. Tak *Yes*
2. Nie *No*
3. Nie spotkałem się z lekarzem w ostatnim roku *I haven’t seen a doctor or health professional in the past 12 months*

**2.8 Czy w przeciągu ostatniego roku przestawałeś(aś) palić na jeden dzień lub dłużej ponieważ chciałeś(aś) rzucić palenie?** *During the past 12 months, have you stopped smoking for one day or longer because you were trying to give up smoking?*

1. Tak *Yes*
2. Nie *No*

**2.9 Czy obecnie** *Are you currently:*

1. Próbujesz przestać palić *Trying to quit*
2. Aktywnie planujesz aby przestać *Actively planning to quit*
3. Myślisz o rzuceniu, ale nie planujesz *Thinking about quitting, but not planning to*
4. Nie myślisz o rzuceniu *Not thinking about quitting*

**2.10 Proszę zaznaczyć czy zgadzasz się, nie zgadzasz się lub jesteś niepewny(a) co do następujących stwierdzeń**

Please state whether you agree, disagree or are unsure about the following

| **Jeśli rzucił(a)bym palenie, wierzę, że**  ***If I gave up smoking, I believe that:*** | Zgadzam się  *Agree* | Nie zgadzam się  *Disagree* | Jestem niepewny(a)  *Unsure* |
| --- | --- | --- | --- |
| Mój stan zdrowia polepszyłby się w krótkim terminie  *My health would improve in the short term* |  |  |  |
| Mój stan zdrowia polepszylby się w dłuższym terminie  *My health would improve in the long term* |  |  |  |
| Przybrał(a)bym na wadze  *I would put on weight* |  |  |  |
| Byłoby mi ciężej znosić stres  *It would be harder to handle stress* |  |  |  |
| Czuł(a)bym, że zrobił(a)em coś wartościowego  *I would feel I had done something worthwhile* |  |  |  |

**2.11 Gdzie zwykle nabywasz papierosy?** *From where do you usually get your cigarettes?*

1. Irlandia *Ireland*
2. Polska *Poland*
3. Inne źródła, podaj *Other (please specify) ………*

**2.12 Ile płacisz za paczkę 20 papierosów?** *How much do you usually pay for a pack of 20 cigarettes?*

______złotych *Zloties*

_______ euro *Euro*

**Każdy kto zostawi swoje dane teleadresowe do wykorzystania jedynie w celach marketingowych przez firmę 123 Happy Media Ltd i Research Institute for a Tobacco Free Society otrzyma drogą pocztową lub przez email Polski Miesięcznik SOFA a także weźmie udział w losowaniu jednego z 5 bezpłatnych, 1-miesięcznych kursów języka angielskiego metodą Callana w Szkole Gordon & Gordon w Dublinie.** *Everyone who provides us with mobile phone number, email, postal address and name will receive a free copy of the Polish Lifestyle Magazine SOFA and will take part in drawing 1 of 5, 1-month long free English language courses Callan Method in Gordon and Gordon English language school.*

Komórka *mobile* Email Imię i nazwisko *name* Adres pocztowy *postal address*

Ending time of interview Date of interview
