# Supplementary material for: Smoking characteristics of Polish immigrants in Dublin
Source: BMC Public Health. 2008 Dec 31;8:428. doi: 10.1186/1471-2458-8-428 (PMC2630949; doi:10.1186/1471-2458-8-428)
Supplement: Additional file 2 — Questionnaire 2. Survey questionnaire in English. [file 1471-2458-8-428-S2.doc]

**Demographics**

1. What is your gender?

1. Male
2. Female

2. What age are you? _____ yrs

3. What is the highest level of education you have completed to date?

1. Some primary (not complete)
2. Primary or equivalent
3. Intermediate/junior certificate or equivalent
4. Secondary/ leaving certificate or equivalent
5. Diploma/ certificate
6. Primary degree
7. Postgraduate/ higher degrees

4. What is your current marital status?

- 1. Single (never married)
  2. Cohabiting
  3. Married
  4. Seperated
  5. Divorced
  6. Widowed

5. Which of the following descriptions best describes your usual situation with regard to work?

1. Employee (including apprentice or Community Employment)
2. Self employed (not including farming)
3. Farmer
4. Student Full time
5. On state training scheme (FAS, Failte Ireland etc)
6. Unemployed, actively looking for a job
7. Long term sickness or disability
8. Home duties /looking after the home or family
9. Retired (please specify last occupation before retirement)_________________________________________________
10. Other (please specify)________________________________________

6. What is your approximate level of net household income? This means the total income, after tax and PRSI, of ALL MEMBERS of the household. It includes ALL TYPES of income: income from employment, social welfare payments, child benefit, rents, interest, pensions etc. We would just like to know into which broad group the total income of your household falls.

| Per week (€) | Per month (€) | Per year (€) | Tick |
| --- | --- | --- | --- |
| Under 193 | Under 834 | Under 10000 |  |
| 193 - 384 | 834 - 1667 | 10000-19999 |  |
| 385 – 575 | 1668 - 2500 | 20000-29999 |  |
| 576 - 767 | 2501 - 3333 | 30000-39999 |  |
| 768 - 959 | 3334 - 4167 | 40000-49999 |  |
| €960 or more | 4168 or more | 50000 or more |  |

7. In what country were you born?

1. Ireland
2. Poland
3. Other (please specify)

8. If born in Poland, when did you arrive in Ireland? (Month/Year) __________________

9. In total, how long do you plan to live in Ireland?

1. 0 to 6 months
2. 6 to 12 months
3. 13 to 18 months
4. 19 – 24 months
5. More than 2 years

**Tobacco Use**

1. Have you yourself smoked at least 100 cigarettes (5 packs of 20) in your lifetime?

1. Yes
2. No (if No, then skip the rest of tobacco use section)

2. Have you ever smoked daily for six months at least?

1. Yes → If yes, how old were you when you started to smoke daily? ______ years
2. No

3. How often do you currently smoke?

1. Everyday
2. Somedays
3. Not at all

If you answered a) Everyday or b) Somedays, please skip to question 6 through 12.

If you answered c) Not at all, please answer question 4 and 5.

**This section is for ex-smokers only**

4. **If you do not currently smoke**, how long has it been since you last smoked?

1. Within the past month (anytime less than 1 month ago)
2. Within the past 3 months (1 month but less than 3 months ago)
3. Within the past 6 months (3 months but less than 6 months ago)
4. Within the past year (6 months but less than 1 year ago)
5. Within the past 5 years (1 year but less than 5 years ago)
6. Within the past 10 years (5 years but less than 10 years ago)
7. 10 or more years ago

5. **If you do not currently smoke**, what age were you when you quit? _____years old

**The next section is for current smokers only**

6. **If you currently smoke,** how many cigarettes do you usually smoke per day? ____

7. In the past 12 months did a doctor or health professional discuss ways of giving up smoking with you?

1. Yes
2. No
3. I haven’t seen a doctor or health professional in the past 12 months

8. During the past 12 months, have you stopped smoking for one day or longer because you were trying to give up smoking?

1. Yes
2. No

9. Are you currently?

1. Trying to quit
2. Actively planning to quit
3. Thinking about quitting, but not planning to
4. Not thinking about quitting

10. Please state whether you agree, disagree or are unsure about the following

| **If I gave up smoking, I believe that:** | Agree | Disagree | Unsure |
| --- | --- | --- | --- |
| My health would improve in the short term |  |  |  |
| My health would improve in the long term |  |  |  |
| I would put on weight |  |  |  |
| It would be harder to handle stress |  |  |  |
| I would feel I had done something worthwhile |  |  |  |

11. From where do you usually get your cigarettes?

1. Ireland
2. Poland
3. Other (please specify)
4. 12. How much do you usually pay for a pack of 20 cigarettes?

______Zloties

_______ Euro
